# The Pathogenesis of Rift Valley Fever

**DOI:** 10.3390/v3050493

**Published:** 2011-05-06

**Authors:** Tetsuro Ikegami, Shinji Makino

**Affiliations:** 1 Department of Pathology, The University of Texas Medical Branch, 301 University Blvd. Galveston, TX 77555, USA; 2 Department of Microbiology and Immunology, The University of Texas Medical Branch, 301 University Blvd. Galveston, TX 77555, USA; E-Mail: shmakino@utmb.edu; 3 The Sealy Center for Vaccine Development, The University of Texas Medical Branch, 301 University Blvd. Galveston, TX 77555, USA; 4 The Center for Biodefense and Emerging Infectious Diseases, The University of Texas Medical Branch, 301 University Blvd. Galveston, TX 77555, USA

**Keywords:** Rift Valley fever virus, pathogenesis, hemorrhagic fever, encephalitis, blindness

## Abstract

Rift Valley fever (RVF) is an emerging zoonotic disease distributed in sub-Saharan African countries and the Arabian Peninsula. The disease is caused by the Rift Valley fever virus (RVFV) of the family *Bunyaviridae* and the genus *Phlebovirus*. The virus is transmitted by mosquitoes, and virus replication in domestic ruminant results in high rates of mortality and abortion. RVFV infection in humans usually causes a self-limiting, acute and febrile illness; however, a small number of cases progress to neurological disorders, partial or complete blindness, hemorrhagic fever, or thrombosis. This review describes the pathology of RVF in human patients and several animal models, and summarizes the role of viral virulence factors and host factors that affect RVFV pathogenesis.

## 1. Introduction

Rift Valley fever (RVF), a mosquito-borne zoonotic disease among humans and ruminants, is caused by Rift Valley fever virus (RVFV) belonging to family *Bunyaviridae*, genus *Phlebovirus* [1,2]. RVF is endemic to sub-Saharan African countries and has caused major outbreaks in several countries including Kenya, Tanzania, Somalia, South Africa, Madagascar, Egypt, Sudan, Mauritania, Senegal, Saudi Arabia, and Yemen [3]. Pregnant ruminants infected with RVFV typically are subject to high-rate abortions, fetal malformation, and subclinical-to-fatal febrile illness, while newborn lambs usually die by acute hepatitis [4–6]. RVFV infection in humans primarily causes a self-limiting febrile illness; however, some patients develop hemorrhagic fever, neurological disorders, or blindness after the febrile period [5,7,8]. In endemic area, floodwater Aedes mosquitoes, such as *Ae.mcintoshi* or *Ae.vexans,* serve as vectors, and the virus could be transmitted into offspring transovarially [9,10]. Heavy rainfall or flooding of river banks due to construction of dams increases the number of permanent fresh water species of mosquitoes such as *Culex pipens*, which play a role in amplifying RVFV among mosquitoes, ruminants and humans [10–15]. An outbreak of RVF in developed countries, e.g., the U.S. or Europe, could force a curtailing of livestock movement to prevent RVFV spread, causing massive economic loss, and a substantial degree of panic in our society, because the body fluids of infected animals contain infectious RVFV [16,17], and mosquitoes such as Culex spp. Aedes spp. or Anopheles spp. might further spread RVFV into other mosquitoes, humans and animals [18–20]. Effective vaccines and antiviral drugs are necessary for the containment of outbreaks and treatment of RVF patients, respectively. However, neither safe and effective vaccines nor efficient treatment is available. A correct understanding of RVF pathogenesis is essential for the development of effective vaccines and antiviral drugs against RVF. In this review, we will describe clinical and pathological findings of RVF in humans and animals and discuss viral and host factors that affect RVF pathogenesis.

## 2. Pathogenesis of RVF in Humans and Animals

### 2.1. Human

**Figure 1:**
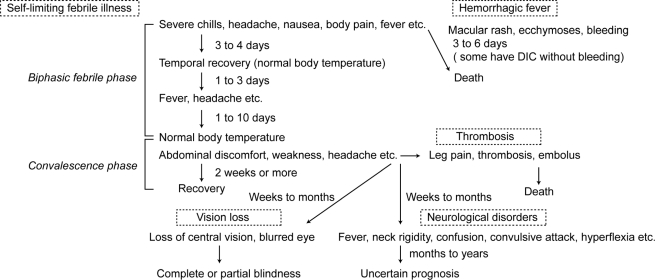
The pathological forms of Rift Valley fever in humans.

Most RVF patients suffer from a self-limiting, febrile illness. However, some patients develop neurological disorders, vision loss, hemorrhagic fever, or thrombosis as shown in Figure 1.

#### 2.1.1. Self-Limiting Febrile Illness

In the 1930s–40s, many RVFV laboratory infections occurred due to a lack of appropriate biosafety procedures [21–25]. However, the patients in most of these and later outbreaks suffered from self-limiting and nonfatal illness [4,6,22,23,25–28]. Typically, the incubation period for RVF is 4 to 6 days. Symptoms start abruptly with severe chills, malaise, dizziness, weakness, severe headache, nausea and/or sensation of fullness over the liver region [6,22,23]. These symptoms are followed by an elevated body temperature (38.8 °C to 39.5 °C); decreased blood pressure; pain in the back, shoulders, neck or legs; rigor; shivering; flushed face; red eye with sore; constipation; insomnia and/or photophobia. Occasionally, other symptoms are seen which include epistaxis, abdominal pain, lack of gustatory discrimination, vomiting and/or diarrhea [4,6,22,25,26,28]. Some lessening of symptoms can be observed on the 3rd day, and the body temperature often decreases to a normal level by the 4th day after the onset of symptoms. However, within 1 to 3 days after the recovery of body temperature, some patients again experience a temporal recurrence of high fever with a severe headache for a few days [6,25,26]. Moreover, patients may have a long-lasting high fever for as much as 10 days [6]. After body temperature becomes normal, some patients may develop a massive coronary thrombosis [26], persistent aching of legs for two weeks [4,22], or persistent abdominal discomfort for weeks [4]. The palpable enlargement of the liver and spleen is not common. In the convalescence phase, patients often experience weakness, malaise, a tendency to sweat, frequent headaches, pain on motion of the eye, and a sense of imbalance. Virus has been demonstrated in the blood during the febrile period (3–4 days), whereas neutralizing antibody also starts appearing around the 4th day of the onset of symptoms [6,22,24,25,27].

#### 2.1.2. Neurological Disorders

Maar *et al*. described a case of encephalitis in a RVF patient [29]. The patient exhibited symptoms of sudden fever, rigor, and retro-orbital headache for two days. He had fever again at the 22nd day after the onset of illness and experienced neck rigidity lasting for five days from the 25th day. Subsequently, he was sometimes confused and otherwise mentally affected, and experienced temporal vision loss without detectable retinopathy. He also exhibited convulsive attacks, hyperflexia and fever until the 50th day. His serum contained anti-RVFV hemagglutination (HAI) antibodies of 1:160 at the 25th day and 1:640 at the 40th day, while his cerebrospinal fluid (CSF) contained 1:2 of HAI antibody at the 28th day and 1:64 at the 50th day. The CSF also contained an increased number of white blood cells consisting mainly of lymphocytes at the 28th day, indicative of the possible occurrence of viral meningitis or meningoencephalitis. The patient recovered after treatment with amantadine, rifampicin, and dexamethasone for two weeks, although the effect of therapy could not be evaluated precisely.

Another case with encephalitis and retinitis was described by Alrajhi *et al.* [30]. The patient had a fever, ataxic gait, and bilateral retinal hemorrhage. She could not count fingers, and the CSF contained many leukocytes, including lymphocytes. Her consciousness level was decreased. She was discharged on day 30 of the illness to her home, at which time she was awake, blind, quadreparetic, and incontinent. Moreover, her neurologic conditions did not improve for the next year.

An additional report described a patient who had persistent hemiparesis for four months after the onset of illness [31], and another paper reported 12 RVF patients, who developed neurological signs and symptoms, including meningeal irritation, confusion, stupor and coma, hypersalivation, teeth-grinding, visual hallucinations, locked-in syndrome, and choreiform movement of upper limbs [32]; in these patients, the histopathological lesions in brains were characterized by focal necroses associated with an infiltration of round cells, mostly lymphocytes and macrophages, and perivascular cuffing [32].

#### 2.1.3. Vision Loss

Some patients suffer from maculopathy or retinopathy. Patients noticed the loss of central vision or blurred eye occurring at various times after infection; e.g., from immediately after the disease onset to several weeks or months later. One or both eyes could be affected [33–35], and the affected eyes had macular edema with exudates containing a white mass covering the macular area with or without retinal hemorrhage, vasculitis, infarction or vitreous haze [34–39]. In addition, retinal detachment [35,36], uveitis [38,39], or arterial occlusion [35,36,39–41] was reported in some patients. In many cases, a complete recovery of vision does not occur, and chorioretinal scarring can remain in macular and paramacular areas, in spite of the resorption of exudates [34,35,37–41], while some patients show partial improvement in vision after several months of RVFV infection [34,36,38,40].

#### 2.1.4. Hemorrhagic Fever

Fatal RVF cases often involve hemorrhagic manifestations but the time to death varies among cases [42–44]. Most typically, the illness starts suddenly, and the patients experience fever, rigor, nausea, vomiting, headache, injected conjunctives, drowsiness, and/or body pains. The patients may also have such symptoms as macular rash over the entire trunk, ecchymoses on the arms, limbs, and/or eyelids, bleeding from the gums and/or gastrointestinal mucosal membrane, low blood pressure, hematemesis, melena, diarrhea, throat pain, pneumonitis, jaundice, and/or hepatosplenomegaly [42,43]. Typically, elevation of alanine aminotransferase (ALT), aspartate aminotransferase (AST), lactate dehydrogenase (LDH), and reduction of platelet count and hemoglobin are seen in these patients [42,45]. In many cases, death occurs in 3 to 6 days after patients become symptomatic; however, in some cases, death occurs in 12 to 17 days after the onset of symptoms. Postmortem examination shows diffuse necrosis of hepatocytes which more greatly affect the centrilobular area than the portal area, which may indicate association with acute hepatic injury in this type of pathogenesis [42,44]. It should be noted that some patients who do not exhibit jaundice or hemorrhage, die from renal failure or disseminated intravascular coagulation (DIC) accompanied by an elevation of ALT, AST, LDH or D-Dimer, or a decrease in platelet count [46,47]. A group of RVF patients who died from typical hemorrhagic fever also had encephalitis in addition to hepatic and gastro-intestinal necroses [32], which demonstrates the neuroinvasiveness of RVFV in hemorrhagic patients.

#### 2.1.5. Thrombosis

Another type of fatal case of RVFV infection was described by Schwentker *et al*. [21]. They reported that the temperature of the patient fell to normal on the 4th day after the onset of symptoms, whereas two papular areas of several centimeter in diameter were found on the patient’s thigh and leg on the 5th day and remained until day 8. After temporal recovery by the 12th day, the patient experienced phlebitis of the popliteal vein, which was followed by infarcts in the lungs on the 20th, 26th and 34th days at multiple locations; these eventually caused a fatal embolus in the pulmonary vessels on the 45th day of illness. The liver of the patient was normal, did not contain infectious RVFV, and no typical RVF lesions were confirmed at the postmortem histopathological examination. However, there was a large thrombus in the inferior vena cava, a part of which might have detached and caused an embolus in the pulmonary artery. The neutralizing antibody showed up on the 6th day of illness, and its titer increased toward the 12th day.

#### 2.1.6. Possible Vertical Infection

In a retrospective study in Egypt, no increases in the total number of abortions were seen during an RVF outbreak, and the serological conversion rate of aborted women before and after outbreak was 31.1% and 27.5%, respectively [48]. A report describing a potential vertical infection of RVFV concerned a pregnant woman, who experienced fever, headache, dizziness and generalized muscle ache four days before delivery during the RVF outbreak in Saudi Arabia in 2000 and developed IgG specific to RVFV [49]. Her newborn baby presented with an anti-RVFV IgM antibody, as well as ALT/AST elevation, jaundice, extension of the activated partial thromboplastin time (APTT: test for the deficiency of intrinsic pathway factors) and the prothrombin time (PT: test for the deficiency of extrinsic pathway factors), and died on the 6th day after birth [49]. Although it is unknown whether the newborn baby died from RVF, it is possible that a vertical transmission *in utero* might have occurred in this case.

The clinical symptoms of RVF vary among patients. The determinant of host susceptibility to induce hemorrhagic fever in humans has not been characterized. It is also unknown how RVFV causes diseases such as neurological disorders, vision loss or thrombosis in the presence of protective antibodies. Several animal models have been used to understand the pathology of RVF, and the advantages and disadvantages of different animal models are summarized in Table 1. We discuss the pathological findings in various different animal models in the next chapter.

### 2.2. Mouse

Mice are one of the most susceptible animal species to RVFV infection [6,8], and RVF pathology in infected mice mimics the pathological findings in newborn lambs [6]. Most of the mice infected with wild-type (wt) RVFV ZH548 or ZH501 strains die in 3 to 5 days [50–52], whereas they die faster by infection with other wt isolates [6,53]. Infected mice show ruffed fur with decreased activity in 2 to 3 days, and then become more lethargic while lying with their back legs wide apart [6,52]. The symptom is often followed by death within one hour [6]. Occasionally, mice survive this stage, yet have hind limb paralysis at days 8–9 post infection (p.i.) and die from encephalitis [52]. The rectal temperature of infected mice is often normal or decreased to below normal [54]. Also, the clotting time of blood derived from RVFV-infected mice is significantly extended, and it clots normally by mixing with normal sera, a finding that may indicate the shortage of coagulation factors is important for the extension of clotting time [54].

The liver is the major target organ of RVFV, while the enlargement of liver is not common [6,52]. Liver lesions are characterized by fulminant hepatitis, with coagulative necroses leaving the portal space intact [50,52,55,56]. Depletion of glycogen in hepatocytes is also common at an early stage [6,51,55]. Infected hepatocytes in mice contain eosinophilic intranuclear inclusion bodies [6,52], which are not reactive to the Feulgen reaction, a nucleic acid stain [57]. The intranuclear inclusion bodies in RVFV-infected cultured cells were visualized by an indirect immunofluorescent assay with antisera against RVFV and found to have a filamentary shape [58]. Inclusion bodies are formed by the NSs protein [59], a viral nonstructural protein, and the 10-to-17 amino acids at the carboxyl terminus of NSs are responsible for the formation of the filamentous structures via self-association [60]. Viral antigens start accumulating in hepatocytes at day 2, and their abundance increases extensively at day 3 p.i. [52]. The infected hepatocytes are stained by using a terminal deoxynucleotidyl transferase dUTP nick end labeling (TUNEL) assay, indicating that RVFV replication induces apoptosis in hepatocytes [52]. Mice that survived the early hepatitis phase often are able to regenerate hepatocytes [52,61]. Although swollen endothelial cells can be observed in the liver [6], antigens are not easily detectable in endothelial cells or Kuppfer cells, which indicate that these cells are not the primary targets of RVFV [52,55].

In addition to hepatocytes, viral antigens have been detected in, odontogenic and gingival epithelium; lipocytes; pituicytes; olfactory neurons and multiple types of neurons in the brain; mononuclear phagocytes; cardiac myofibers; and in perineural, periosteal, adrenocortical, endosteal, perivascular, bone marrow stromal, fibroblastic reticular, and vascular smooth muscle cells, as well as in cells morphologically consistent with dendritic, pancreatic islet, and adrenal medullary cells; however, no viral antigens were reported in any ocular structure, including the retina [52]. Apoptosis of lymphocytes were found in the thymus, spleen, lymph nodes and mucosa-associated lymphoid tissues [52]. Congestion and hemorrhage are common to the liver, spleen, lymph nodes, large intestine, kidneys and brain [6,52], but are uncommon in the jejunum [54]. In some mice that survived acute viral hepatitis, a sharp decrease in viral antigens occurred at 8 days p.i., and no virus could be detected in the sera, liver, lung, pancreas, large intestine and ovaries [52]. In the late stage of infection, however, lethal meningoencephalitis characterized by neuronal necrosis, microhemorrhages, and perivascular cuffs occurs in mice that survived the acute hepatitis [52].

### 2.3. Rat

The susceptibility of rats to RVFV differs among rat strains [62]. Peters *et al*. demonstrated that 10-to-15-week-old inbred rats from U.S. breeders exhibited three different responses to subcutaneous (s.c.) RVFV inoculation [63,64]. Wistar-Furth (WF) and Brown Norway strains were highly susceptible to RVFV and died within four days p.i. by liver necrosis, while the Fisher 344, Buffalo, DA and Lewis strains were largely resistant to RVFV infection [64]. ACI and Maax strains proved to be moderately susceptible and showed ascending paralysis; lesions were mainly in the brain and spinal cord and characterized as mild-to-severe necrotizing encephalitis and encephalomyelitis with focal necrosis with neutrophilic infiltrate and perivascular cuffing primarily with lymphocytes. In the ACI and Maax strains, viruses were undetectable in the liver and blood, whereas 5-to-6 log pfu/g of viruses could be detected from brain tissue, even in the presence of neutralizing antibody in the serum. The intracranial injection of RVFV uniformly caused encephalitis in these rats, including the resistant Lewis strain. The immunosuppression of the resistant Lewis rats by treating animals with cyclophosphamide 1 day prior to s.c. RVFV infection resulted in death at around 5-to-7 days p.i. with increased viral titers in the serum, liver, spleen, brain, kidneys and adrenal gland, although the virus titers in these organs in the Lewis rat were still lower than those in the corresponding organs of the WF strain [65]. These data suggest that the Lewis rat encodes a gene(s) important for the resistant phenotype. Interestingly, WF (WF/mol) and Lewis rats (Lewis/mol) obtained from a European breeding colony are resistant and susceptible to RVFV, respectively; hence, these rats showed the opposite susceptibilities to RVFV infection to those of the same strains from U.S. breeders. Furthermore, both WF and Lewis rats obtained from another European breeder were resistant to RVFV infection; taken together, these findings indicate the possible genetic variability of inbred rats among different breeders [66]. Cross-breeding experiments culminated in findings that indicated the resistance of WF/mol rat was segregated as a single Mendelian dominant locus [66]. Findlay *et al*. also showed that the albino rat of the Glaxo strain had an age-dependent susceptibility to RVFV via the i.p. route infection [62]; rats younger than 15 days died in 2 to 4 days with extensive liver necrosis, whereas 26-day-old rats survived RVFV infection [62].

### 2.4. Hamster

The Syrian hamster is one of the most susceptible rodents to RVFV. Death occurs in 2 to 3 days p.i. after intraperitoneal inoculation with massive liver necrosis [6,67]. The pathological changes are similar to those seen in mice [6]. Administration of low titers of neutralizing antibodies protects hamsters from fatal liver necrosis, yet infected hamsters die from encephalitis by day 11 [67].

### 2.5. Gerbil

The gerbil, *Meriones unguiculatus*, represents a unique RVFV animal model, which produces fatal encephalitis with minimal liver involvement after infection of non-neuroadapted wt RVFV. The gerbil has proven moderately susceptible to RVFV, and the survival rate of 10-week-old gerbils after s.c. inoculation is reported to range from 50 to 100%, dependent on the strain and inoculation dose [68]. Death was reported to occur around 1 to 3 weeks in a dose-independent manner; s.c. inoculation of 10^7^, 10^5^, 10^3^, 10^1^ pfu of ZH501 resulted in 90%, 100%, 60% and 60% survival of outbred Tum:(MON) gerbils, respectively, and 50%, 90%, 70% and 70% survival of inbred MON/Tum gerbils, respectively [68]. The infected gerbils exhibited hind-limb paralysis, generalized weakness and wasting. Gerbils also showed an age-dependent resistance to RVFV infection. Most of the 3- to 5-week-old Tum:(MON) gerbils died after 10^7^ pfu s.c. inoculation of ZH501 from encephalitis, whereas 90% of 7-week-old gerbils were able to survive the infection. After s.c. inoculation, RVFV replicated temporally in the livers of both 4-week-old and 10-week-old gerbils on days 1 and 2 (∼10^3^ pfu/g), while subsequent efficient virus replication in the brain occurred in 4-week-old gerbil (from day 4 to day 7 up to 10^7^ pfu/g), but not in 10-week-old gerbil (temporal increase up to 10^2^ at day 7) [68]. Intracerebral wt RVFV inoculation of 50 pfu into 10-week-old gerbils resulted in an efficient viral replication (∼10^7^ pfu/g) in the brain and the mean time to death was six days, which is not statistically different from that of 4-week-old gerbils, which may indicate the presence of host factors influencing the neuroinvasiveness in an age-dependent manner [68]. Histopathologically, minimal multifocal necroses of hepatocytes are seen at days 1 or 2 after the s.c. inoculation of wt RVFV, while focal necrotizing encephalitis with neuronal necrosis, a neutrophilic infiltrate, and perivascular cuffing are seen in the brain at later time points [68]. Mild, necrotizing encephalitis without detectable infectious RVFV could be observed even in clinically normal RVF-infected gerbils [68].

### 2.6. Nonhuman Primates

Rhesus macaques are moderately susceptible to RVFV infection [6]. After i.p or intranasal inoculations, body temperatures of infected macaques increased to 39–40 °C at 1 to 4 days p.i. and the febrile period lasted for 24 to 120 h, whereas some infected animals did not show any febrile reactions [6]. Peters *et al*. first described hemorrhagic fever-like illness in rhesus macaques that were experimentally infected with a wt RVFV ZH501 strain [69]. Three out of fifteen rhesus macaques intravenously inoculated with RVFV ZH501 became ill; two became moribund and were euthanized on days 7 and 3, and one recovered from illness, whereas the others showed temporal viremia, the maximum viral titer of which occurred around day 2, and were clinically normal. All three monkeys exhibited lassitude, weakness, the cessation of food intake, petechiae, ecchymoses and bleeding from nares, gums or venipuncture sites. Clear extensions of APTT, slight extension of PT, and a decrease in the number of platelets were observed in the two dead monkeys, possibly indicating a deficiency of coagulation factors and platelets. Histopathologically, the dead monkeys showed moderate focal or midzonal coagulative necrosis of the liver involving approximately 1/3 to 2/3 of hepatocytes, necrosis in the ventricular myocardium, fibrin thrombi in the glomeruli and small intertubular vessels of renal medulla in the kidneys, and mild depletion of lymphocytes from white pulp and the deposition of eosinophilic amorphous fibrin-like material in red pulp cords in the spleen [69].

Morrill *et al*. reported that after intravenous inoculation of 1 × 10^5^ pfu ZH501 strain into 17 rhesus macaques, three developed signs of hemorrhagic fever, seven were clinically ill but survived, and the other seven survived without clinical signs [70]. Serum interferon (IFN)-α was detected from 6–24 h p.i. and from 24–30 h p.i. in the surviving monkeys and in those dying, respectively, and the delayed IFN response was preceded by viremia in two of the three lethally-infected monkeys [70]. No surviving monkeys developed signs of encephalitis or retinal complications in follow-up observations at two months to two years [70]. Morrill *et al*. also demonstrated that the administration of recombinant leukocyte A IFN (5 × 10^5^ U, i.m) at 6 h after RVFV intravenous inoculation reduced the peak viremia titer by 100-times and cleared viruses by 48 h p.i. [71]. These studies suggested the importance of IFN-α in limiting viral replication.

Findlay *et al*. reported that three species of African monkey, *i.e.*, the green guenon (*Cercopithecus callitrichus*), the sooty mangabey (*Cercocebus fuliginosus*) and the Patas guenon (*Erythrocebus patas*), did not exhibit any febrile reaction after inoculation of RVFV, whereas virus was detected in the blood [72]. In contrast, four species of South American monkeys, two brown capuchin monkeys (*Cebus fatuellus* and *Cebus chrysopus*) and two common marmosets (*Callithrix jacchus* and *Callithrix penicillata*) exhibited febrile reactions for 1 to 2 days upon RVFV infection, which may indicate that South American monkeys are more susceptible to RVFV infection than African monkeys [72]. Davies *et al*. reported that RVFV-infected baboons (*Papio Anubis*) had viremia for 3 to 4 days without developing significant clinical signs [73].

### 2.7. Sheep

Daubney *et al.* originally reported an outbreak of RVF in a herd of sheep in Kenya in 1930, which was characterized as a high rate of abortion in pregnant ewes and high mortality of newborn lambs [4]. A later study by Easterday *et al*. described that the mortality of adult sheep following experimental RVFV infection was approximately 20% [74]. Typically, sheep with more than one week old were relatively resistant to RVFV infection, yet did exhibit fever (39 to 40 °C), viremia, diarrhea, nasal discharge, and decreased activity [74,75]. Nine- to ten-week-old young adult sheep (Ripollesa breed) that were subcutaneously inoculated with RVFV had corneal and choroidal edema with inflammatory infiltrate, which could be associated with drainage failure or inadequate corneal dehydration after transient viremia [76]. On the other hand, 7- to 11-month-old Yansaka sheep subcutaneously inoculated with RVFV died during the viremic febrile phase and displayed symptoms of epistaxis (2 days p.i.∼), severe and bloody diarrhea, conjunctival hemorrhage, widespread petechiae and ecchymoses in hairless areas, pulmonary edema/hemorrhage, and thrombi formation in the blood vessels of the heart, kidneys and brain. RVFV-infected West African Dwarf or the Ouda breed did not exhibit such rapid hemorrhagic symptoms and rather exhibited marked coagulative hepatic necrosis, and brain lesions, including mild gliosis, neural degeneration, neurophagia, and satellitosis [77]. Interestingly, Yansaka, West African Dwarf and Ouda also had increased prothrombin time, which may have indicated that hemorrhage was induced by a combination of vascular endothelial damage and an inability to clot blood in response to the damage [77]. The inconsistency of symptoms and mortality in adult sheep described in these publications suggest the divergence of host genetic background, even within the same breed of sheep, affects susceptibility to RVFV infection.

Several studies examined the effects of RVFV vaccine candidates for protecting pregnant ewes from wt RVFV infection. However, insufficient immunogenicity of inactivated vaccines or residual virulence of live-attenuated vaccines induced fetal malformations. Pregnant ewes were untreated (n = 8) or vaccinated (n = 50) once with formalin-inactivated RVFV and then challenged with wt RVFV ZH501 at 45 days post vaccination [78]. Abortion occurred in both unvaccinated and vaccinated ewes, between 6 to 18 days p.i., and 50% of the ewes aborted their fetuses. One out of 50 vaccinated ewes and one out of eight unvaccinated ewes died. These data may indicate that the inactivated RVFV vaccine induced an insufficient immunity for sheep. Necropsy at 19 days p.i. revealed that 100% of the ewes had either aborted or dead fetuses, while the dead or aborted lambs showed extensive liver necrosis typical of RVF [78]. Coetzer *et al*. reported that immunization of pregnant ewes with a live-attenuated Smithburn vaccine strain at 42 to 74 days of pregnancy could cause a teratogenic effect in the fetus, including arthrogryposis, hydranencephaly, or mineralization of brain with or without *hydrop amnii*, and two out of six lambs from the nine vaccinated ewes showed such effects [79]. Hunter *et al*. described that pregnant ewes inoculated with live-attenuated MP-12 vaccine strain at 28 to 56 days of gestation either miscarried or produced lambs showing teratogenic effects (11 out of 75 lambs from 50 vaccinated ewes), such as cerebellar hypoplasia, spinal hypoplasia, hydranencephaly, prognathia inferior, brachygnathia inferior, arthrogryposis, scoiliosis, lordosis, kyphosis, or dormed head [80]. The abortion and teratogenous effects did not occur when pregnant ewes were vaccinated with MP-12 at the third trimester of pregnancy, *i.e.*, at 90-110 days of gestation [81,82]. On the other hand, pregnant ewes vaccinated at 15 days of gestation with a RVFV Clone 13 (C13) strain, which has a 69% in-frame deletion of NSs [83], did not cause abortion or fetal malformations and were protective against wt RVFV challenge [84]. These data may imply the involvement of MP-12 NSs in the abortion of ewes and the teratogenic effects in lambs.

RVFV infection causes an acute and fatal disease in newborn lambs [5]. RVFV-infected newborn lambs usually exhibit obvious illness, including elevated body temperature (40 to 41 °C), loss of appetite, decreased activity, and prostration, about 12 to 18 h prior to death [85]. The mortality rate in RVFV-infected newborn lambs is 95 to 100% [5]. Studies of experimental infection of 1-4 day-old lambs with RVFV via s.c. resulted in necrosis of isolated hepatocytes (12–18 h p.i.), focal coagulative necrosis of hepatocytes (24–33 h p.i.), and extensive hepatocyte necrosis (48–51 h p.i.) with a progressive increase in viral antigens, whereas no viral antigens could be detected in the endothelial or Kupffer cells in the liver, suggesting that hepatocytes are the primary target of RVFV [86]. The necrosis is predominantly centrilobular or midzonal, and yet there is no definite distribution pattern in liver necrosis [5,87]. Some infected lambs also exhibited necrosis in the villi at the distal jejunum and ileum and depletion of lymphocytes in the spleen, whereas the brain and eyes had no lesions [87]. Overall, the liver pathology of newborn lambs resembles that of mice or hamsters, which are extremely susceptible to RVFV. However, the RVFV neurovirulence in lambs is less prominent when compared with that in rodents.

RVFV also causes diseases in other animals including goats, cattle, camels, dogs, cats, and ferrets, but does not cause any symptomatic diseases in rabbits, guinea pigs, birds, horses, pigs and other animals, as reviewed in detail previously [4–6,8,63,88–93]. As of the present, the mechanism of species-specific susceptibility to RVFV infection is unknown.

RVFV infection shows unique pathogenesis in each animal model. Because viral replication and host antiviral responses most probably contribute to viral pathogenecity, an understanding of RVF pathogenesis requires identification and characterization of the viral virulence factors and host antiviral factors. The next chapter describes the viral determinant of virulence.

## 3. Viral Determinants of Virulence

### 3.1. Virus Life Cycle

**Figure 2:**
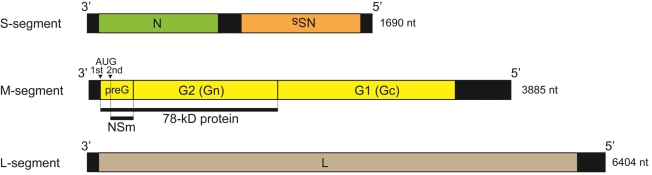
Schematic representation of Rift Valley fever virus (RVFV) genome structure. S-encodes N and NSs proteins in an ambisense manner, the M-segment NSm, 78 kD protein Gn and Gc, and the L-segment L proteins. The 78 kD and NSm proteins are synthesized from 1st and 2nd AUG of M mRNA.

The RVFV genome is comprised of three RNA segments named the S-, M- and L-segments (Figure 2) [1,2]. The S-segment encodes N and NSs genes in an ambisense manner, the M-segment. NSm (NSm2), 78 kD (NSm1), Gn and Gc genes, and the L-segment, the RNA-dependent RNA polymerase (L) gene [10]. RVFV virions bind to an unidentified cellular receptor, and enter the cells in a pH-dependent manner [94], probably through a clathrin-mediated endocytic pathway, as described for another phlebovirus [95]. After viral uncoating, viral ribonucleocapsid (RNP) composed of viral genomic RNA segments and N protein [96] is released into the cytoplasm, and the viral polymerase, which probably is attached to the RNP exerts primary transcription to synthesize viral mRNA [97]. Both N mRNA and NSs mRNA are transcribed during primary transcription as early as 40 min after infection from an efficiently packaged, viral-sense (negative-sense) S-segment and anti-viral-sense (positive-sense) S-segment, respectively; the packaging mechanism of RVFV RNP and the presence of specific *cis*-signals in the genomic RNA are not known [97]. Viral RNA replication starts around 1 to 2 h after infection [97] and an increase in the amount of viral genomic RNA results in increases in viral mRNAs and proteins. The RNP is packaged into viral virions probably by its interaction with the cytoplasmic domains of Gn/Gc at the Golgi apparatus, as reported for other bunyaviruses [98,99]. The three different RNA segments could be co-packaged in a coordinated manner, in which the co-packaging of M and S-segments could support the packaging of the L-segment [100]. The RVFV virion surface is highly symmetric T = 12 icosahedral lattice [101], which is formed by a shell of 122 glycoprotein capsomers most probably composed of 720 Gn-Gc heterodimers [102].

### 3.2. Role of NSm Protein in Viral Virulence

The RVFV M-segment encodes 78 kD, NSm, Gn and Gc proteins in M mRNA (Figure 2). Those proteins are synthesized from a single open reading frame of M mRNA at different AUGs present at the 5′ region of M mRNA by leaky scanning of ribosomes, while the N-terminus of Gn and Gc is co-translationally cleaved by host proteins [103–105]. NSm is synthesized from the 2nd AUG, and the C-terminus is generated by cleavage at the N-terminus of Gn, while the 78 kD protein is synthesized from the 1st AUG, and the C-terminus is identical to that of Gn. Among seven viral proteins, NSs and NSm are nonstructural proteins which are not incorporated into virions [59,104]. The 78 kD protein has not been studied in detail, while an “80 kD” protein induced by RVFV infection (most probably corresponding to the current 78 kD protein) is known to be incorporated into virions [106], which may indicate that the 78 kD protein is a structural protein. NSs, 78 kD protein and NSm are dispensable for viral replication in cell cultures [83,107–109]. Infection of 12-week-old female WF rats with a recombinant RVFV ZH501 strain lacking both 78 kD protein and NSm induced acute fatal hepatic disease, causing the deaths of some infected rats around four days p.i., or a delayed fatal neurologic disease, resulting in death of some of infected animals around 13 days p.i. (mortality rate: 50 to 70%), while wt ZH501-infected rats developed acute hepatitis and 100% died. These data suggest that NSm is not essential for virulence and lethality [110]. On the other hand, a recombinant RVFV MP-12 lacking the expression of both 78 kD and NSm induced more extensive apoptosis than did MP-12 in cultured cells and the expression of NSm significantly inhibited the cleavage of caspase-8 and -9 induced by staurosporine [111], demonstrating that NSm protein suppresses apoptosis. Thus, NSm probably suppresses apoptosis in infected hosts and affects viral pathogenicity.

### 3.3. Role of NSs Protein in Viral Virulence

NSs is known to be a major virulence factor of RVFV; a RVFV C13 strain, which has a 69% in-frame deletion of NSs [83], and is completely attenuated in mice or sheep [51,84]. Further studies showed that NSs inhibits the synthesis of IFN-β mRNA under the conditions that transcription factors, such as IFN regulatory factor 3 (IRF-3), NF-κB and activator protein (AP)-1, are activated in wt RVFV ZH548-infected cells [112]. It was found that NSs can bind and sequester the p44 subunit of TFIIH, an essential transcription factor for RNA polymerase I and II; and hence NSs was reported to prevent the assembly process of the TFIIH complex, resulting in the suppression of host mRNA transcription [113]. Le May *et al*. also described studies demonstrating that a region of NSs, corresponding to amino acid 210 to 230, specifically binds to Sin3A-associated protein (SAP30) and forms a complex that represses the histone acetylation required for the transcriptional activation of IFN-β promoter; this repression worked even after the binding of IRF-3 to the IFN-β promoter [114]; hence, recombinant ZH548 carrying a deletion at amino acid 210 to 230 of NSs cannot suppress the IFN-β mRNA synthesis. It should be noted that C13 NSs bound to SAP30, yet it did not suppress IFN-β expression [114]. Although the inability of C13 NSs to suppress IFN-β expression may have been due to its poor accumulation in infected cells, further studies are required to know how the binding of NSs to SAP30 can lead to the suppression of IFN-β mRNA synthesis. It is also uncertain whether the general host transcriptional suppression is required for the inhibition of IFN-β mRNA synthesis.

Because NSs induces host general transcription suppression [113], RVFV might have the ability to replicate under host transcription suppression. Actinomycin D (ActD) is a general inhibitor of host RNA synthesis. Viral titers of several cytoplasmic RNA viruses including arenaviruses [115–117], measles virus [118], sindbis virus [119], rubella virus [120], polio virus [121,122], coronaviruses [123,124], and lactic dehydrogenase elevating virus [125] could be reduced in the presence of ActD, while the viral RNA synthesis is often unaffected [115,118,123]. We found that expression of NSs protein is essential for the RVFV MP-12 strain to actively synthesize viral proteins and produce high titers of infectious viruses in the presence of ActD [126]; cells infected with recombinant RVFV MP-12 lacking NSs failed to synthesize viral proteins; and while cells infected with MP-12 lacking NSs accumulated phosphorylated eIF2α. The latter made cellular translation initiation inactive through the activation of dsRNA-dependent protein kinase (PKR) at around 8 h p.i. [126], resulting in the suppression of viral protein synthesis. Further studies revealed that MP-12 NSs promoted the degradation of PKR through the proteasome pathway and prevented an accumulation of phosphorylated eIF2α, thereby securing efficient viral protein synthesis under host transcription shut-off induced by ActD [126]. Habjan *et al*. reported that wt RVFV NSs also induced PKR degradation and demonstrated that an RVFV C13 strain carrying biologically inactive NSs induced fatal hepatic disease in C57BL/6 mice lacking PKR [127]; these mice are competent for inducing type-I IFNs in response to viral RNA replication or poly (I):poly (C) [128]. PKR is one of several IFN-stimulated genes (ISGs) and plays an important role in inhibiting viral replication *in vivo*; other RNA viruses, including vesicular stomatitis virus (VSV) and influenza virus, also replicate more efficiently in mice lacking PKR than in those with an intact PKR [129]. In addition to PKR, IFN-induced MxA proteins is also known to inhibit RVFV replication [130].

Although the genetic diversity of RVFV strains is relatively low (approximately 5% in primary sequences [131]), the susceptibilities to rat IFN-α/β differed among RVFV strains. Most of the sub-Saharan RVFV strains are very sensitive to rat IFN-α/β (ED_50_: 0.3–0.7 units), whereas Egyptian strains, including ZH501 and ZH548, and a Zimbabwean isolate (2269/74) are relatively resistant to rat IFN-α/β (ED_50_: 50–200 units) [132]. All of those RVFV isolates show a similar sensitivity to human IFN-α (ED_50_: 70–880 units).

In summary, RVFV NSs induces the shut-down of host transcription, including transcription of both type-I IFN and ISGs mRNAs, to prevent antiviral responses. NSs also induces the degradation of PKR to prevent eIF2α-mediated host and viral translational shut-off and promote an efficient viral protein synthesis.

### 3.4. Other Virulence Factors

Although NSs is a major virulence factor to escape host innate immune responses, the virulence of RVFV could be controlled in a polygenic manner. The RVFV MP-12 strain is a highly attenuated strain derived from wt RVFV ZH548 [133] and encodes a functional NSs gene. Mutations in the M- and L-segments are major determinants of MP-12 attenuation [134,135]. In contrast, the C13 strain encodes an S-segment lacking a functional NSs gene, while the M- and L-segments of C13 strain are still virulent phenotypes [51,83]. Mice lacking IFN-AR (IFN-AR^−/−^ mice) are susceptible to both MP-12 and C13, while the viral replication kinetics of MP-12 and C13 differ in those mice; the highest titer of viremia was reached within 28 h p.i. and around 48 h p.i. in C13-infected mice and in MP-12-infected mice, respectively [51]. Thus, there is a possibility that M- and L-segments of C13 may facilitate a rapid replication of C13 in these mice, reaching the highest virus titer substantially earlier compared to MP-12-infected mice. Recently, we found that wt RVFV ZH501 virus stock contains two major viral populations, rZH501-M847-A (Glu at aa.123 of Gn protein) and rZH501-M847-G (Gly at the corresponding site); the difference in the amino acid is mapped within one of the neutralizing epitopes in Gn protein [61,136]. Although it is not known how the two different populations have emerged in the ZH501 virus stock, which was amplified once in the mouse brain and passaged twice in FRhL cells and twice in Vero E6 cells, the rZH501-M847G replicated less efficiently than rZH501-M847A in infected mice, whereas both of them replicated efficiently in tissue cultures such as MRC-5, VeroE6, J774.1, and NIH3T3 cells. Our study showed that one amino acid change in the Gn can substantially alter replication and pathogenesis of ZH501 *in vivo*, and yet the mechanism of the Gn mutation in the pathogenesis remains unknown. Clearly further studies will be needed for understanding the roles of viral proteins in RVFV pathogenesis.

RVFV encodes several virulence factors, and the major virulence factor NSs plays an important role in evading host innate immune responses. In the next chapter, we discuss how humans or host animals develop protective immune responses against highly virulent wt RVFV.

## 4. Host Determinants of Virulence

### 4.1. Host Defense via Infection Route

Adequate immunity against RVFV can attenuate the virulence of RVFV in animals and vaccination against RVFV can save animals from lethal RVFV challenge [84,133,137–140]. The passive transfer of neutralizing antibodies is sufficient for protection from lethal RVF [67,141–144], whereas the role of the cellular immune response for the protection is not sufficiently evaluated. Yet, Mandell *et al*. demonstrated that mice immunized with virus-like particles containing N have a higher survival rate (survival: 9/16) than those not containing N after lethal RVFV challenges (survival: 3/16). Because anti-N protein antibody is unlikely to neutralize RVFV, these data may point to the involvement of cellular immune responses against N protein for RVFV protection [145]. Alternatively, antiviral immune responses might be induced by forming a complex between anti-N antibody and N proteins released from dead cells or infected cells *in vivo* [146]. If N proteins are present in cell surface as reported in influenza virus-infected cells [147,148], complement-mediated cell lysis could be another mechanism to support the elimination of infected cells [146].

Aerosol exposure is one of the most likely routes for both laboratory infections and bioterrorism attack. Immunization of rats by s.c. inoculation of a formalin-inactivated RVFV vaccine (TSI-GSD-200) partially protected lethal RVFV aerosol exposure at 187 days post immunization (survival: 72/105: 69%), whereas 11 out of 72 surviving rats developed encephalitis without clinical signs at 27-28 days post-challenge, which was revealed during necropsy [149]. Another study showed that three vaccinated rats challenged with RVFV aerosol developed uveitis, although no histopathological analysis was presented [141]. Mice are highly susceptible to wt RVFV aerosol exposure (LD_50_: 1 to 2 PFU), which may cause lethal hepatitis, but not pneumonia [150]. A study in mice vaccinated at several different routes with formalin-inactivated RVFV vaccine (NDBR-103) [149] and challenged with RVFV subcutaneously showed that the immunization route affected survival rates; s.c. immunization, intraperitoneal (i.p.) immunization, and intraduodenal (i.d.) immunization resulted in survival rates of 97.5%, 100% and less than 20%, respectively. Another study reported that fewer than 20% of mice immunized via s.c. or i.d. and about 50% of mice immunized via i.p. survived after aerosol route challenge of RVFV in the 2-week observation period [151]. Findings from this study using aerosol RVFV challenge indicated that the mucosal immunity elicited by i.d. immunization successfully lowered the occurrence of olfactory bulb encephalitis, but failed to prevent necrotic hepatitis, and immunity induced by s.c. or i.d. did not prevent hepatitis, olfactory bulb encephalitis and multifocal encephalitis, while i.p. immunization completely prevented the occurrence of hepatitis, but not multifocal encephalitis [151]. These reports seem to indicate that immunization with inactivated vaccines via s.c. cannot prevent RVFV-induced diseases after aerosol challenge. It will be important to establish a reliable countermeasure against bioterrorism by use of RVFV through further detailed characterization of RVFV replication in various organs after aerosol challenge and examination of the efficacy of immunization of current live-attenuated RVFV vaccine candidates, such as MP-12 or C13, for preventing RVF-induced diseased after RVFV aerosol challenge.

Exposure of animals to aerosol containing RVFV probably results in initial RVFV infection in lung epithelial cells, such as type I alveolar epithelial cells. Both infection and release of RVFV occur in polarized epithelial cells, such as Caco-2 cells (human colorectal adenocarcinoma cells), at apical and basolateral membranes [152]. Infection by Punta Toro virus (PTV), which belongs to the *Phlebovirus* genus and causes a lethal necrotic hepatitis in hamsters [153,154] and mice [155], results in virus being released into the basolateral membrane [156], which may contribute to systemic viral spread in infected animals.

### 4.2. Host Susceptibility

As described in Section 2.3, several studies point to the possibility that unidentified host genetic factors influence the susceptibility of inbred rat species to RVFV. The primary rat hepatocytes derived from resistant American Lewis rats or WF/mol rats are less permissive to RVFV infection than those derived from susceptible American WF rat or Lewis/mol rats [66,157], whereas RVFV replicates efficiently in primary cortical glial cells derived from WF/mol rats [66] or spontaneously transformed cell lines generated from embryonic thymus cells of either resistant LEW rats or susceptible WF rats [132], suggesting that hepatocytes in those resistant inbred rats are under the control of a host factor(s) that restricts efficient RVFV replication. Peritoneal macrophages (PM) derived from resistant Lewis rat or susceptible WF rat were treated with 1.0 or 10 U of IFN, which resulted in a 150- or 3300-fold reduction of ZH501 replication in Lewis PM, and a 4- or 250-fold reduction in WF PM, respectively, possibly indicative of the role of IFN to increase resistance in Lewis rats [158]. On the other hand, primary hepatocytes derived from susceptible Lewis/mol rats and resistant WF/mol rats are resistant to RVFV after treatment of the cells with rat type-I IFN, suggesting the resistance induced by type-I IFN is not necessarily important for the host specific resistance seen in WF/mol rats [66].

A recent study showed that MBT/Pas mice, but not BALB/cByi mice, are highly susceptible to RVFV ZH548 infection [159]. Experiments using microarray and quantitative real-time PCR showed that RVFV ZH548 replication in mouse embryonic fibroblast (MEF) cells derived from susceptible MBT/Pas mice induced higher levels of Ifnb1 and Ifna4 mRNAs than did those derived from resistant BALB/cByj mice, while MEF cells derived from MBT/Pas mice failed to induce several ISGs, including the IFN regulatory factor 7 (Irf7) mRNA, the 2′-5′ oligoadenylate synthetase-like 2 (Oasl2) mRNA, and the IFN-induced 17 kDa protein (Isg15) mRNA [159]. The knockout of Isg15 or Oasl2 mRNA expressions increased viral replication in MEF cells derived from resistant BALB/cByj mice, leading the authors to suggest that MBT/Pas mice have a defect in their IFN responses which controls RVFV spread [159]. It is of interest to know whether the mice lacking Isg15 or Oasl2 genes are susceptible to RVFV. It is unknown whether the susceptibility of inbred rat to RVFV is due to a deficit in some ISGs.

The host factors determining the host susceptibility to PTV infection have been explored; PTV infection in mice mimics RVFV-induced lethal hepatitis. C57BL/6J mice show an age-dependent susceptibility to PTV infection; the mice gradually become resistant to PTV from 5 to 7 weeks, and they are resistant at eight weeks of age [155]. The 8-week-old C57BL/6J mice show a delayed viremia, when compared to 4-week-old mice, and the peak viremia titers in the 8-week-old mice are 5- to 10-times lower than those in the 4-week-old mice [155]. Likewise, PTV replication was lower in primary cultured hepatocytes, Kupffer cells and peripheral blood monocytes isolated from 8-week-old C57BL/6 mice than in those cells obtained from 3-week-old C57BL/6 mice [160,161]. Adding stress to the 8-week-old mice by daily handling and observation increased their susceptibility to PTV replication [162]. The role of toll-like receptor (TLR) 3, which is a pathogen recognition receptor recognizing double-stranded RNA, in the susceptibility of the mice to PTV was studied by using 8-week-old TLR3^−/−^ mice of the C57BL/6 background [162]. The wt mice infected with PTV had a 100% mortality and high levels of IL-6 induction (yet no remarkable increase in TNF-α), whereas mice lacking TLR3 infected with PTV had increased survival rates, slightly earlier reduction of serum virus titers, and decreased levels of serum IL-6 [162]. On the other hand, IL-6^−/−^ mice were more susceptible to PTV infection and supported higher levels of viremia than did wt mice, which possibly means that IL-6 is indispensable for protective immunity. A similar attenuation of virulence has been also reported for West Nile virus infection in TLR3^−/−^ mice [163]. The authors hypothesized that over-production of IL-6 in wt mice would be detrimental to the outcome of PTV infection [162], pointing out that the balance of cytokines might alter the pathogenesis of PTV. STAT-1 is a key molecule in the IFN signaling pathway [164]. It was reported that PTV replicated substantially better in the brains of STAT-1^−/−^ mice than in wt mice. Also PTV titers in sera, spleens and livers of the STAT-1^−/−^ mice were high, which may emphasize the importance of IFN responses for limiting viral replication in the liver, spleen and brain [165].

## 5. Conclusions

Since the first recognition of RVF in an outbreak in 1930, more than 80 years has passed. Although there has been good progress in characterizing clinical, pathological, and virological features of RVFV infection, RVFV still causes outbreaks in African countries or the Arabian Peninsula [3,10]. The development of effective, safe, highly immunogenic and economic vaccines for animals and humans will prevent RVF in the endemic countries [137]. There are several important questions to address in controlling RVFV and in further understanding RVF pathogenesis. Some of them include determining the: (1) mechanism that triggers hemorrhagic fever, (2) route of viral entry to the brain, (3) mechanism of prolonged diseases in RVF patients in the presence of neutralizing antibodies, and (4) significance of vaccination to prevent RVF after aerosol exposure. Addressing these questions will have a substantial impact on understanding of RVF pathogenesis and development of anti-RVFV reagents.

## Figures and Tables

**Table 1:** Advantages and disadvantages of animal models for Rift Valley fever.

**Model**	**Advantages**	**Disadvantages**
Mouse	Highly susceptible to RVFV Infected mice usually die in 2 weeks, and are suitable for RVFV challenge study Acute hepatitis and lethal meningoencephalitis at late stage Cost-effective	No hemorrhagic fever No ocular diseases
Rat	Varied susceptibility among inbred strains Suitable for studying host genes responsible for RVFV-resistant phenotype Similar pathological changes to those in mice A report suggests the presence of uveitis after aerosol challenge Cost-effective	The inbred strains of same name derived from different breeding colonies have different susceptibility to RVFV. Age-dependent difference in susceptibility
Hamster	Highly susceptible to RVFV Similar pathological change to those seen in mice Often used for experimental RVFV transmission by mosquitoes	No hemorrhagic fever No ocular diseases Limited research resources
Gerbil	Encephalitis with minimum liver diseases Useful for studying neuroinvasiveness	No significant diseases except for Encephalitis Age-dependent difference in susceptibility Limited research resources
Rhesus monkey	Lethal hemorrhagic fever Similar susceptibility to humans Important for testing the safety of vaccines or antivirals before clinical trial	No ocular diseases reported Less than 20% develop hemorrhagic fever Requirement of ABSL4 or BSL3+ space to keep monkeys Expensive
Adult sheep, ewe	A report suggests the occurrence of hemorrhagic fever and edema of corneal and choroidal edema with inflammation High rate of abortion and fetal malformation Suitable for veterinary vaccine study	Susceptibility varies among different breeds Requirement of ABSL4 or BSL3+ for large animals Limited research resources Expensive
Lamb	Highly susceptible to RVFV Lethal acute hepatitis Important to evaluate the effect of collostrum from vaccinated ewes	Neurovirulence is not prominent No hemorrhagic fever No ocular diseases Requirement of ABSL4 or BSL3+ for large animals Limited research resources
